# The associations between staffing hours and quality of care indicators in long-term care

**DOI:** 10.1186/s12913-018-3552-5

**Published:** 2018-10-03

**Authors:** Veronique M Boscart, Souraya Sidani, Jeffrey Poss, Meaghan Davey, Josie d’Avernas, Paul Brown, George Heckman, Jenny Ploeg, Andrew P Costa

**Affiliations:** 1 Schlegel Centre for Advancing Seniors Care, 299 Doon Valley Drive, Kitchener, ON N2G 4M4 Canada; 2 Conestoga College Institute of Technology and Advanced Learning, Doon Campus, Rm 2A220, 299 Doon Valley Drive, Kitchener, ON N2G 4M4 Canada; 3 Ryerson University, 350 Victoria St, Toronto, ON M5B 2K3 Canada; 4 University of Waterloo, 200 University Avenue West, Waterloo, ON N2L3G1 Canada; 5 Schlegel-University of Waterloo Research Institute for Aging, 250 Laurelwood Dr, Waterloo, ON N2J 0E2 Canada; 6 Schlegel Villages, 325 Max Becker Dr, Kitchener, ON N2E 4H5 Canada; 7 McMaster University, 1280 Main Street West, Hamilton, ON L8S 4L8 Canada

**Keywords:** Long-term care, Nursing homes, Nursing assistants, Quality of care, Health care aides

## Abstract

**Background:**

Long-term care (LTC) staffing practices are poorly understood as is their influence on quality of care. We examined the relationship between staffing characteristics and residents’ quality of care indicators at the unit level in LTC homes.

**Methods:**

This cross-sectional study collected data from administrative records and resident assessments from July 2014 to June 2015 at 11 LTC homes in Ontario, Canada comprising of 55 units and 32 residents in each unit. The sample included 69 registered nurses, 183 licensed/registered practical nurses, 858 nursing assistants, and 2173 residents. Practice sensitive, risk-adjusted quality indicators were described individually, then combined to create a quality of care composite ranking per unit. A multilevel regression model was used to estimate the association between staffing characteristics and quality of care composite ranking scores.

**Results:**

Nursing assistants provided the majority of direct care hours in LTC homes (76.5%). The delivery of nursing assistant care hours per resident per day was significantly associated with higher quality of resident care (*p* = < 0.01). There were small but significant associations with quality of care for nursing assistants with seven or more years of experience (*p* = 0.02), nursing assistants late to shift (*p* = < 0.01) and licensed/registered practical nurses late to shift (*p* = 0.02).

**Conclusions:**

The number of care hours per resident per day delivered by NAs is an important contributor to residents’ quality of care in LTC homes. These findings can inform hiring and retention strategies for NAs in LTC, as well as examine opportunities to optimize the NA role in these settings.

**Electronic supplementary material:**

The online version of this article (10.1186/s12913-018-3552-5) contains supplementary material, which is available to authorized users.

## Background

According to the most recent national health statistics available (2014 in the United States, 2011 in Canada), long-term care (LTC) homes across North America provided medical care and activities of daily living (ADL) support to over 1.6 million people [1, 2]. Many of these residents have complex medical conditions as well as frailty and depend on staff assistance for their daily activities [3–5]. When a resident is more dependent for assistance with activities of daily living, sufficient care time and staff competency are required to achieve quality care standards [6–8].

There are different approaches to assess care quality; Donabedian’s framework is one of the most widely accepted [9, 10]. This framework categorizes quality of care indicators into structure (i.e. organizational factors), process (i.e. care delivery actions), and outcome (i.e. patient) components [9, 10]. In LTC settings, the most frequently cited resident quality care indicators are patient outcomes. Examples include pressure ulcers, functional status, mortality, hospitalization, nutrition status and incontinence [10–13].

Sufficient and competent staffing is critical although the amount of care time delivered varies regionally. In the United States, federal legislation (i.e. Nursing Home Reform Act (1987)) has considerably low minimal standards for staffing: it requires *sufficient staff* to meet resident needs and one registered nurse Director of Nursing for eight hours a day for seven days a week and a licensed nurse in evening and night shifts [14]. State legislation varies but tends to have higher staffing standards; although, this has also been criticized by experts as inadequate [14]. According to the 2016 national report for LTC homes in the United States, staff time varies by facility type and resident case mix: the average of registered nurse (RN) hours per resident per day (hprd) ranged from 0.27 to 0.32, the average of licensed practical or vocational nurse (LPN) hprd ranged from 0.16 to 0.34, and the average of nursing assistant (NA) hprd ranged from 1.71 to 2.76 for a total number of 2.14 to 3.42 hprd [15]. In Canada, long-term care is offered through a mix of public, private for-profit, private not-for-profit, and religious-based providers. All LTC homes are legislated and funded by the province (although accommodation costs are shared as co-payments with residents). Provinces operate under the Canada Health Act (1984) which lists conditions required for federal funding of public health care services [16]. Across Canada, an RN on duty who is present at all times is generally required and provinces may additionally require a minimum number of paid staff hours per resident per day (e.x. Alberta requires 1.90 paid hprd from nursing and personal services staff whereas Ontario makes no such stipulations) [17, 18].

Several studies have investigated the associations of staff time with residents’ quality of care in LTC, however, because of limitations in study design and power, there remains uncertainty. Increases in RN staff time are associated with improvements in several quality of care domains (i.e., decreased physical restraints, pressure ulcers, catheterization, urinary tract infections, ADL decline, nutritional supplements, hospitalizations, and improved resident satisfaction) [10, 13, 19]. Increases in L/RPN care hours is associated with decreased pressure ulcers, ADL decline and physical restraint use [20]. Increases in NA care time is associated with decreased infections, pressure ulcers, ADL decline, incontinence, and physical restraint use [20].

Despite promising findings, the true benefits of high staffing levels in LTC are uncertain due to low data quality, insufficient study power, unknown mechanisms of action, and disparate measurement of quality and LTC home characteristics [10–13]. Additionally, existing LTC staffing practices are poorly understood: while LTC homes are required by law to provide minimum RN hours (which may vary by state or province), other nursing and support services are provided at the discretion of the LTC home on an as-needed basis, making these services highly variable [21, 22].

The purpose of this study was to explore the association between the number of staff care hours per resident per day and residents’ outcomes. The hypothesis investigated was that higher hours of direct care for residents is associated with better quality of care outcomes.

## Methods

We performed a cross-sectional study across a chain of 19 for-profit LTC homes in Ontario, Canada from July 1, 2014 to June 30, 2015. Data were available from 11 LTC homes, representing 55 total units and 32 residents in each unit, serving approximately 5000 residents and employing 5738 staff members. The study sample included all existing and newly admitted LTC residents, as well as all RN, L/RPN and NA staff (both full-time and part-time, as well as agency-provided) employed at these facilities. The unit of analysis was the 55 units clustered within 11 LTC homes. This study was approved by the relevant Research Ethics Boards.

### Sources of data

We collected data from the organization’s staffing records as well as provincially-mandated resident assessment data for the eleven LTC homes. The staffing data included staff hours worked per day, agency staff hours per day, minutes late to shift, minutes worked past shift end, number of years worked at the current LTC home and staff employment status (full-time, part-time, casual). The number of hours staff worked per day is calculated from the data collected by a scanner system where all staff are required to “punch-in” and “punch-out” at the beginning and end of their shift. This information was collected daily by the human resources department and de-identified records where shared.

Data on residents’ clinical characteristics, case mix, and outcomes were collected from the provincially-mandated Resident Assessment Instrument –Minimum Data Set (RAI-MDS) 2.0. RAI-MDS is a comprehensive assessment completed at admission, quarterly, and annually, and when a significant change in health status occurs for each resident in all LTC homes in Ontario [23]. This assessment was implemented in all US nursing homes in 1996 and is currently used in most provinces of Canada, as well as Europe, Asia, and Pacific Rim [23, 24]. We chose the risk adjusted quality indicators from RAI-MDS because these indicators are mandatory to report in Canadian and US nursing homes [25, 26]. We collected all resident assessments during the study period. We reported on the following RAI-MDS scales: depression rating scale (DRS) (i.e., a clinical screen for depression); changes in health, end-stage disease and signs and symptoms (CHESS) (i.e., a measure of health instability); pain (i.e., presence and intensity of pain); ADLs; cognitive performance scale (CPS) (i.e., a measure of consciousness, executive function and memory); and aggressive behavior scale (ABS) [23]. In addition, we reported on 13 practice sensitive RAI-MDS 2.0 quality indicators, which are considered to be responsive to clinical practice changes, including: pressure ulcer, worsening pain, physical restraint use, antipsychotic use without psychosis, indwelling catheter, delirium, declining behavioral symptoms, urinary tract infections, late loss ADL decline, fallen in the last 30 days, feeding tube, decline in mood, and unexplained weight loss [27]. Less than 1% of the population had a feeding tube; this item was dropped from the quality of care composite ranking score.

### Statistical analyses

Descriptive analyses included means and standard deviations (SD) for data with a normal distribution (i.e., resident characteristics and staffing characteristics) and medians and interquartile ranges (IQR) for skewed data (i.e., quality indicators). We defined care hours per resident per day by total hours worked, divided by the sum of each day’s number of residents on each unit. Staff hours per resident per day was accrued to each unit and averaged over the 1-year timeframe. Staff time for staff working across multiple units (i.e., RNs, L/RPNs) was attributed across units.

We chose to investigate associations of staff time with a quality of care composite ranking. According to configurational theory, no single outcome can be expected to account for the complexity of managing organizations (such as a LTC home) [28]. Indeed, when a unit performs well in some areas and more poorly in others, it is challenging to conclude the overall effects of staff time in that unit. A more intuitive approach is to rank units on their performance on individual quality indicators and then create an overall summary ranking score. To define an overall measure of quality of care, we created a quality of care composite ranking for each unit similar to that of the US Centers for Medicare and Medicaid’s Five-Star system [29]. First, we adjusted the quality indicators using Canadian standard algorithms, equivalent to those used by the Canadian Institute for Health Information [30]. Across the 55 units, we assigned quintile values of 1 (poorest quality) to 5 (highest quality) to each practice sensitive RAI-MDS QI, and averaged these quintile rankings.

We applied a multi-level regression model, adjusting for clustering of units within LTC homes, to examine possible associations between staffing characteristics and overall quality of care for units. Using the PROC MIXED procedure, data at the unit were represented as clusters within LTC homes to account for the potential correlation between quality of care composite rankings within LTC homes. Such clustering would violate the assumption of independence of observations. We included the RUG-III case mix index (a widely used measure of expected relative staff time in LTC) to adjust for unit-level variation in staffing demands based on resident care needs [31]. Care hours per resident per day, proportion of shifts late by ten+ minutes, proportion of shifts worked past shift end, NA years of experience in the current home and NA employment status were defined a-priori and entered into the model. All analyses were performed using Statistical Analysis Software (SAS® 9.4, SAS Institute Inc., Cary, North Carolina).

## Results

### Sample size

The sample included 11 homes, 55 units, 858 NAs, 183 L/RPNs, 69 RNs, and 2173 unique residents for the period July 1, 2014 to June 30, 2015.

### Resident characteristics

Resident characteristics are described in Table 1. Most residents were female (68.9%) and over 65 years of age (90.7%). Dementia (66.9%), impaired ADL functioning (52.3%), impaired cognition (63.1%), aggression (58.5%), bladder incontinence (68.7%), and dependent on wheelchair for mobility (58.3%) were common. Fewer residents had mood symptoms (29.7%), severely aggressive behaviors (18.6%), unstable health (21.8%) and wandering behaviors (24.7%).

**Table 1:** Resident Characteristics

	Mean (SD)
Female (%)	68.9 (9.8)
Age (years)	82.7 (2.9)
Age < 65 years (%)	9.3 (8.5)
Alzheimer’s disease and related dementias (%)	66.9 (24.9)
Activities of daily living impairment^a^ (%)	52.3 (12.0)
Cognitive impairment^b^ (%)	63.1 (23.9)
Depressive symptoms^c^ (%)	29.7 (20.1)
Mildly aggressive behaviors^d^ (%)	58.5 (21.4)
Severely aggressive behaviors^e^ (%)	18.6 (13.3)
Unstable health^f^ (%)	21.8 (9.0)
Bladder incontinence daily (%)	68.7 (13.0)
Wandering behaviors (%)	24.7 (16.8)
Wheelchair primarily used for mobility (%)	58.3 (14.9)
RUG-III case mix index	1.098 (0.068)

^a^Defined as a score of 4 or more in Activities of Daily Living Scale (ADL); score range is 0–16

^b^Defined as a score of 3 or more in Cognitive Performance Scale (CPS); score range is 0–6

^c^Defined as a score of 3 or more in Depression Rating Score (DRS); score range is 0–14

^d^Defined as a score of 1 or more in Aggressive Behavior Scale (ABS); score range is 0–12

^e^Defined as a score of 5 or more in Aggressive Behavior Scale (ABS); score range is 0–12

^f^Defined as a score of 2 or more in Changes in Health, End-Stage Disease, and Signs and Symptoms Score (CHESS); score range is 0–5

### Quality of care

The practice sensitive quality indicators are described in Table 2. The most prevalent conditions included mood decline (28.7%), delirium (27.9%), antipsychotic use without psychosis (20.4%), late loss ADL decline (bed mobility, transfers, eating and toilet use) (18.0%), and fall in the last 30 days (18.1%). Indicators which were present in less than 10% of residents included indwelling catheter, urinary tract infection, worsening pain, pressure ulcer, physical restraint use, and unexplained weight loss. The quality of care composite ranking for each unit are displayed graphically as a boxplot in [see Additional file 1: Figure S1].

**Table 2:** Quality Indicators (*N* = 55 units)

	Median (IQR)
Late loss ADL decline^a^ (%)	18.0 (11.4–23.8)
Declining behavioral symptoms (%)	13.1 (7.9–17.5)
Indwelling catheter (%)	1.4 (0.0–4.1)
Urinary tract infection (%)	4.6 (3.0–6.4)
Delirium (%)	27.9 (18.9–31.4)
Antipsychotic use without psychosis (%)	20.4 (13.8–27.6)
Fallen in the last 30 days (%)	18.1 (13.5–21.8)
Mood decline^b^ (%)	28.7 (17.5–37.6)
Worsening pain (%)	5.5 (2.5–7.4)
Pressure ulcer (%)	4.7 (3.1–7.9)
Physical restraint use (%)	8.2 (2.5–10.8)
Unexplained weight loss (%)	9.7 (5.3–13.3)

^a^Defined as a score of 4 or more in Activities of Daily Living Scale (ADL); score range is 0–16

^b^Defined as a score of 3 or more in Depression Rating Score (DRS); score range is 0–14

### Staffing characteristics

Staffing characteristics are described in Table 3. On average, residents received 2.55 h of care per day: 0.15 h were provided by RNs, 0.44 h were provided by L/RPNs, and 1.95 h were provided by NAs. Agency staff contributed approximately 0.01 h of care per resident per day, although this varied widely among units, with many using no agency staffing during the study period. Many NAs had full-time employment status (36.7%) and many worked for at least seven years in LTC (46.3%).

**Table 3:** Staffing Characteristics by Unit (*N* = 55)

	Mean (SD)
Care hours per resident per day	
Registered nurse (RN)	0.15 (0.04)
RN agency staff	0.0032 (0.0055)
Licensed/Registered practical nurse (L/RPN)	0.44 (0.09)
L/RPN agency staff	0.0051 (0.0080)
Nursing assistant (NA)	1.95 (0.24)
NA agency staff	0.0046 (0.0076)
Total	2.55 (0.26)
Late 10+ minutes to shift	
RN (%)	1.7 (1.2)
L/RPN (%)	1.7 (1.3)
NA (%)	1.6 (0.9)
Stayed 30+ minutes past shift end	
RN (%)	27.9 (16.7)
L/RPN (%)	16.2 (8.7)
NA (%)	2.2 (1.3)
Nursing assistant	
Proportion serving 7+ years in the current LTC (%)	46.3 (16.4)
Full-time (%)	36.7 (7.7)

### Model

The association of quality of care ranking with staffing factors within units is described in Table 4, where estimates reflect absolute changes in the average quality of care ranking, after adjusting for all other factors. A unit with one additional NA care hour per resident per day was associated with an improvement (increase) in the quality of care composite ranking score (on a 1 to 5 scale) by 0.89 (p = < 0.01). Controlling for all other factors, a 1 % increase in L/RPNs ‘late to shift’ was associated with an increase in the unit’s quality of care composite ranking by 0.10 (*p* = 0.02), yet a 1 % increase in the percent of NAs late to shift was associated with a decrease of 0.14 in the unit’s quality of care composite ranking (p = 0.02). Additionally, a 1 % increase in NAs with seven or more years of experience in the current LTC home was associated with an increase of 0.01 in the unit’s quality of care composite ranking (p = 0.02). There was no statistically significant association with quality of care for RN care hours, L/RPN care hours, agency staff care hours, the percent of RNs late to shift, any staff staying past shift, or proportion of NAs with full-time employment status. A correlation matrix (See Additional file 2: Table S1) indicates that no correlations exceed .8 and no variable resulted in a variance inflation (VIF) above 10 (multi-variable linear regression with the collinearity diagnostics). There is no evidence multicollinearity was an issue in the model.

**Table 4:** Model of Staffing Effects on Quality of Care Composite Rank (*N* = 55 units)^1,2^

	Estimate	*P*-value
Case mix index	−0.10	0.92
RN hours per resident per day	0.63	0.84
L/RPN hours per resident per day	0.16	0.84
NA hours per resident per day	0.90	< 0.01
Agency staff hours per resident per day	3.50	0.36
RN proportion shifts late 10+ minutes	<−0.01	0.95
L/RPN proportion shifts late 10+ minutes	0.10	0.02
NA proportion shifts late 10+ minutes	−0.14	< 0.01
RN stayed 30+ minutes past shift end	−0.01	0.45
L/RPN stayed 30+ minutes past shift end	0.01	0.68
NA stayed 30+ minutes past shift end	0.03	0.54
NA proportion serving 7+ years in the current LTC	0.01	0.02
NA proportion that are full time	0.01	0.09

^1^Each of 12 quality indicators ranked within 55 units, given a quintile score of 1 (worst) to 5 (best); adjusted for unit nested within the LTC home

^2^Example Interpretation: All things staying the same, a unit with one additional NA hour per patient per day was associated with a quality of care composite ranking score improvement of 0.9 (on the 1 to 5 scale). For variables stated as percentages: All things staying the same, a 1% increase in NA shifts that were started late (by 10 or more minutes) starting their shift 10 min was associated with a quality of care composite ranking decline of 0.14 (on the 1 to 5 scale)

## Discussion

A significant number of older people depend on LTC services [1, 2]. Many studies have attempted to determine the optimal care hours required to meet resident needs but, because of design limitations, there remains uncertainty [10–13].

The characteristics of LTC residents who participated in this study mirrored the provincial data on residents’ age (83 years vs. 82 years provincially), gender (69% female vs. 69% female provincially), diagnosed with dementia (67% vs. 60% provincially) and depressive symptoms (30% vs. 34% provincially) [32]. When compared to data from US nursing homes, residents were similar in terms of gender (69% female vs. 66% female US), ADL impairment (52% vs. 63% US) and cognitive impairment (63% vs. 61% US) [33].

Some quality of care indicators included in this study compared favorably with national and provincial Canadian averages: antipsychotic use without psychosis (20% vs. 23–27% CAN) and worsening pain (5.5% vs. 10.3–10.5% CAN) [27, 29]. The quality of care indicators which did not compare well to the Canadian and provincial (Ontario) averages included falls (18% vs. 15–16% ON and CAN), physical restraint use (8% vs. 6.0–7% CAN), mood decline (29% vs. 22–26% ON and CAN), and pressure ulcers (5% vs. 3% ON and CAN) [32, 33]. The quality indicator rates found in this study were similar with US national reports as well: antipsychotic use (20% vs. 23% US), falls (18% vs. 16% US) and pressure ulcers (5% vs. 5% US) [34]. Based on these estimates, it appears that our sample is broadly representative of North-American LTC resident populations.

According to the most recent report for LTC homes in the United States, the average paid care time in for-profit LTC homes is 0.26 RN hprd, 0.16 LPN hprd and 2.45 NA hprd for a total of 2.87 hprd [15]. However, the report does not distinguish between “worked” hours and “paid hours” (i.e., paid hours could appear inflated because of vacation or sick time). We found that, on average, residents received 88.9% of the reported average number of total care hours, 57.7% of RN care hours, 275.0% of L/RPN care hours, and 79.6% of NA care hours. Decreased care time has been found to be associated with rationing of care which may impact clinical outcomes [35]. While the residents in this study appeared to receive fewer care hours than US national averages, the authors deemed it to be beyond the scope of this study to evaluate if the average care hours are appropriate standards for care. Instead, this study examined the associations of care hours as they relate to residents’ quality of care.

In terms of associations between staffing factors and quality of care, NA care hours were significantly associated with higher overall quality of care; however, there was no such observation with RN or L/RPN hours. A potential reason for this could be that by averaging RN and L/RPN hours per resident per day across the different units per home, we effectively removed any variation at the unit level. However, this was necessary given that RN and L/RPN hours were not assigned to indicusal units. In LTC homes, RN and L/RPN time is mostly dedicated to provide assessment and care only to residents experiencing instability, high complexity and/or are near the end of life. A study focusing specifically on the association between RN and L/RPN time and quality of care for complex residents could observe such an association. Additionally, the numbers of RN or L/RPN staff in this study were low, and may have been dominated by the total staff relationship (RN, L/RPN, NA) with quality of care.

Some of the associations between staffing variables and quality of care were statistically significant, but of a very small size, limiting their clinical relevance. For example, the proportion of L/RPN and NA shifts that were started late (10 or more minutes) were weakly associated with the quality of care composite ranking. Late to shift may represent a complicated combination of staff morale, organization, personal stress, and tangible care available to resident during shift change. The meaning and influence of late to shift might be dependent on a unit’s culture and normal practice patterns. However, none of these inferences could be supported in this study, nor are they supported in the literature. We also found that NAs’ years of experience in the current LTC home was significantly associated with improved overall quality of care. This is likely the result of increased familiarity with residents, families, colleagues, and the organization; as well as being an indicator of low staff turnover, which has been shown to be an important determinant of quality of care [36].

This study used one of the largest samples of LTC residents to date. Additionally, our study described staff hours in detail and related them to a robust quality of care composite ranking, which provides a consolidated view on quality compared to individual quality indicators per unit. However, our study only described one LTC organization and it used a cross-sectional design, which does not establish causality. Despite these limitations, our findings demonstrate the importance of NAs as they provide a meaningful contribution to the quality of care residents receive.

## Conclusions

In conclusion, associations between LTC staffing characteristics and overall quality of care were found for NA care hours and NA years of experience in the current LTC home. This study highlighted the importance of employing and retaining NAs as they contribute to quality of care in LTC homes. There have been smaller studies with similar objectives which showed mixed associations with certain quality of care indicators. To our knowledge, this was the largest multi-site study among nursing home residents and staff which evaluated multiple dimensions of quality of care.

## Additional files

Additional file 1: **Figure S1.** Quality Indicator Quintile Average by Unit. Pictogram showing distribution of quality indicator ranking for units. Note: Scale displays the average quintile values of 1 (poorest quality) to 5 (highest quality) to each practice sensitive RAI-MDS QI. Note: Box plot with jittering (horizontal separation of data points) to show multiple units with the same score. (PDF 46 kb)

Additional file 2: **Table S1.** Correlation Matrix Check for Multicollinearity. Correlation matrix. (PDF 60 kb)

## Abbreviations

ADL: Activities of daily living
L/RPN: Licensed/Registered Practical Nurse
LTC: Long-term care
NA: Nursing assistant
RAI-MDS: Resident assessment instrument-minimum Data Set
RN: Registered nurse
