# Supplementary figures and images for: The associations between staffing hours and quality of care indicators in long-term care
Source: BMC Health Serv Res. 2018 Oct 3;18:750. doi: 10.1186/s12913-018-3552-5 (PMC6171224; doi:10.1186/s12913-018-3552-5)

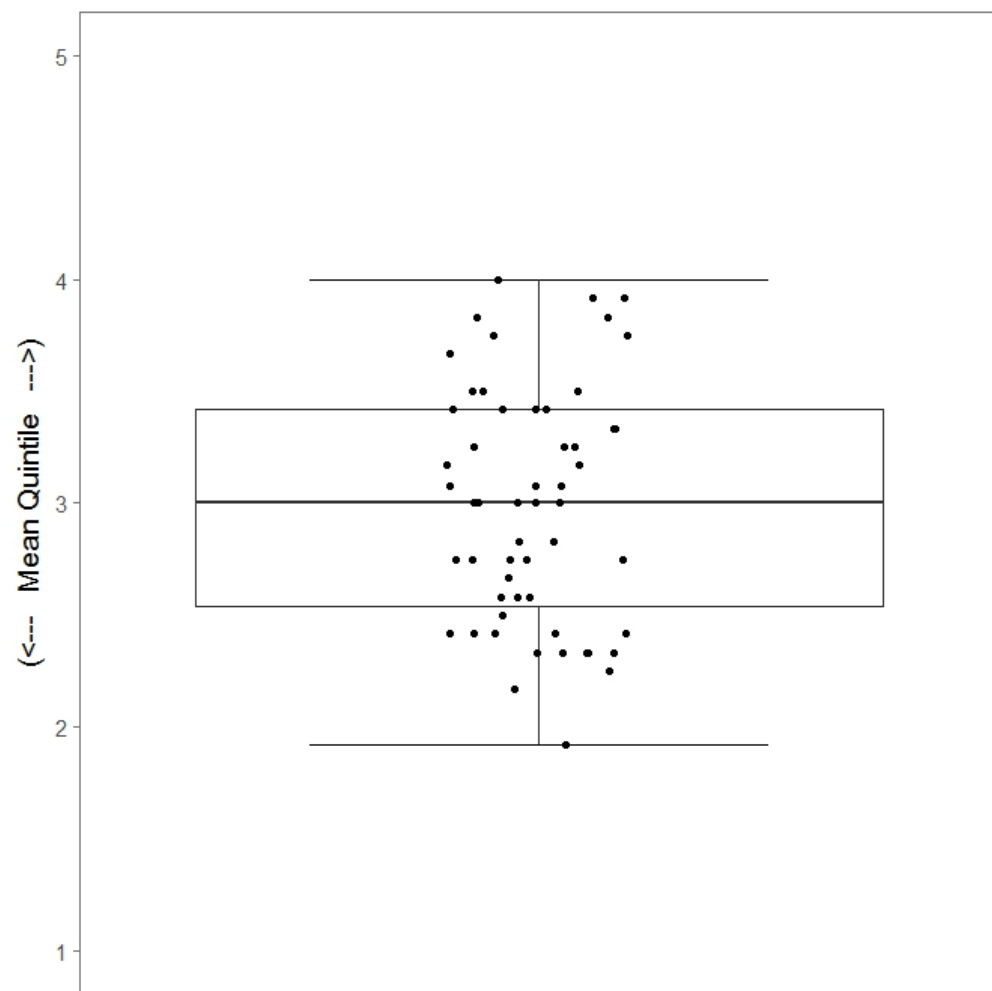

Supplement: Supplementary file 1 — Figure S1. Quality Indicator Quintile Average by Unit. Pictogram showing distribution of quality indicator ranking for units. Note: Scale displays the average quintile values of 1 (poorest quality) to 5 (highest quality) to each practice sensitive RAI-MDS QI. Note: Box plot with jittering (horizontal separation of data points) to show multiple units with the same score. (PDF 46 kb) [file 12913_2018_3552_MOESM1_ESM.pdf]
