# Supplementary material for: The associations between staffing hours and quality of care indicators in long-term care
Source: BMC Health Serv Res. 2018 Oct 3;18:750. doi: 10.1186/s12913-018-3552-5 (PMC6171224; doi:10.1186/s12913-018-3552-5)
Supplement: Supplementary file 2 — Table S1. Correlation Matrix Check for Multicollinearity. Correlation matrix. (PDF 60 kb) [file 12913_2018_3552_MOESM2_ESM.pdf]

NAME	cmi	RN hrs per pt day	RPN hrs per pt day	pca_hrs per pt day	ag_hrs_ptday	rn_late_10mplus_percent	rpn_late_10mplus_percent	pca_late_10mplus_percent	rn_stayed30m_percent	rpn_stayed30m_percent	pca_stayed30m_percent	pca_7years_plus_percent	fulltime_percent
cmi		0.076	0.363	0.402	-0.286	0.003	-0.091	-0.262	0.206	-0.076	0.106	-0.154	-0.270
RN_hrs_per_pt_day	0.076		-0.524	0.150	-0.350	-0.084	-0.507	-0.466	-0.618	-0.522	-0.184	0.386	0.331
RPN_hrs_per_pt_day	0.363	-0.524		0.103	-0.038	-0.128	0.295	0.162	0.433	0.439	0.046	-0.157	-0.380
pca_hrs_per_pt_day	0.402	0.150	0.103		-0.196	-0.238	0.120	-0.003	0.060	-0.219	0.004	-0.011	-0.140
ag_hrs_ptday	-0.286	-0.350	-0.038	-0.196		-0.211	0.062	0.416	0.196	0.324	0.241	-0.449	-0.202
rn_late_10mplus_percent	0.003	-0.084	-0.128	-0.238	-0.211		0.029	0.023	-0.080	-0.131	-0.088	-0.077	0.103
rpn_late_10mplus_percent	-0.091	-0.507	0.295	0.120	0.062	0.029		0.545	0.075	0.215	-0.028	0.211	-0.190
pca_late_10mplus_percent	-0.262	-0.466	0.162	-0.003	0.416	0.023	0.545		0.128	0.370	0.177	-0.050	-0.183
rn_stayed30m_p	0.206	-0.618	0.433	0.060	0.196	-0.080	0.075	0.128		-0.012	0.011	-0.539	-0.246

ercent													
rpn_stayed30m_percent	-0.076	-0.522	0.439	-0.219	0.324	-0.131	0.215	0.370	-0.012		0.349	0.007	-0.428
pca_stayed30m_percent	0.106	-0.184	0.046	0.004	0.241	-0.088	-0.028	0.177	0.011	0.349		0.067	-0.228
pca_7years_plus_percent	-0.154	0.386	-0.157	-0.011	-0.449	-0.077	0.211	-0.050	-0.539	0.007	0.067		0.181
fulltime_percent	-0.270	0.331	-0.380	-0.140	-0.202	0.103	-0.190	-0.183	-0.246	-0.428	-0.228	0.181	
